# Personal

**Published:** 1910-03

**Authors:** 


### PERSONAL.

---

Dr. A. Jacobi, of New York, will soon attain his eightieth birthday. At the recent annual meeting of the Medical Society of the State of New York held at Albany, January 25, 1910, the following resolutions relating to this event were passed:

> That this Society appoint a committee to consider a fitting manner for the celebration of the 80th birthday of that citizen of the world—A. Jacobi.
>
> That this Committee consist of the ex-Presidents of the Society, the incoming President and the ex-Presidents of the State Medical Association.

A meeting of the committee was held at the rooms of the Society in the New York Academy of Medicine Building, February 5, 1910.

---

Dr. D. C. McKenney, of Buffalo, sailed from New York February 19, 1910, for Europe where he will engage in post-graduate work.

---

Dr. John B. Murphy, of Chicago, spent the day February 1, 1910, in Buffalo. He was entertained at luncheon by Dr. Roswell Park, and at dinner by Dr. Marshall Clinton. In the evening he delivered an address before the Buffalo Academy of Medicine on the Surgery of the Joints.

---

Dr. William H. Hodge, of Niagara Falls, was elected President of the Homeopathic Medical Society of the State of New York, at its annual meeting, held at Albany, February 8 and 9, 1910.

---

Dr. A. D. Carmichael, surgeon in charge of the Buffalo station of the United States marine hospital service, has been reappointed for another term of four years at this station, which is considered one of the most desirable in the service.

